# Morpho-Molecular Identification and Pathogenic Characterization of *Fusarium* and *Colletotrichum* Species Associated with Intercropped Soybean Pod Decay

**DOI:** 10.3390/pathogens14101020

**Published:** 2025-10-08

**Authors:** Maira Munir, Muhammd Naeem, Xiaoling Wu, Weiying Zeng, Zudong Sun, Yuze Li, Taiwen Yong, Feng Yang, Xiaoli Chang

**Affiliations:** 1 College of Agronomy, Sichuan Agricultural University, Chengdu 611130, China; mairamunir23@gmail.com (M.M.); wuxl2014@163.com (X.W.); l_yz_deu@163.com (Y.L.); yongtaiwen@sicau.edu.cn (T.Y.); f.yang@sicau.edu.cn (F.Y.); 2 Institute of Crop Science, College of Agriculture and Biotechnology, Zhejiang University, Hangzhou 310058, China; naeem.muhammd@zju.edu.cn; 3 Institute of Economic Crops, Guangxi Academy of Agricultural Science, Nanning 530007, China; zengweiying_1981@163.com (W.Z.); sunzudong639@163.com (Z.S.)

**Keywords:** soybean pods mycoflora, fungal diversity, morpho-molecular phylogeny, pathogenicity assay, disease management

## Abstract

The fruiting stage of soybean (*Glycine max* L.) is critical for determining both its yield and quality, thereby influencing global production. While some studies have provided partial explanations for the occurrence of *Fusarium* species on soybean seeds and pods, the fungal diversity affecting soybean pods in Sichuan Province, a major soybean cultivation region in Southwestern China, remains inadequately understood. In this study, 182 infected pods were collected from a maize–soybean relay strip intercropping system. A total of 10 distinct pod-infecting fungal genera (132 isolates) were identified, and their pathogenic potential on soybean seeds and pods was evaluated. Using morphological characteristics and DNA barcode markers, we identified 43 *Fusarium* isolates belonging to 8 species, including *F. verticillioides*, *F. incarnatum*, *F. equiseti*, *F. proliferatum*, *F. fujikuroi*, *F. oxysporum*, *F. chlamydosporum*, and *F. acutatum* through the analysis of the translation elongation factor gene (*EF1-α*) and RNA polymerases II second largest subunit (*RPB2*) gene. Multi-locus phylogenetic analysis, incorporating the Internal Transcribed Spacer (*rDNA ITS*), β-tubulin (*β-tubulin*), Glyceraldehyde 3-phosphate dehydrogenase (*GADPH*), Chitin Synthase 1 (*CHS-1*), Actin (*ACT*), Beta-tubulin II (*TUB2*), and Calmodulin (*CAL*) genes distinguished 37 isolates as 6 *Colletotrichum* species, including *C. truncatum*, *C. karstii*, *C. cliviicola*, *C. plurivorum*, *C. boninense*, and *C. fructicola*. Among these, *F. proliferatum* and *C. fructicola* were the most dominant species, representing 20.93% and 21.62% of the isolation frequency, respectively. Pathogenicity assays revealed significant damage from both *Fusarium* and *Colletotrichum* isolates on soybean pods and seeds, with varying isolation frequencies. Of these, *F. proliferatum*, *F. acutatum*, and *F. verticillioides* caused the most severe symptoms. Similarly, within *Colletotrichum* genus, *C. fructicola* was the most pathogenic, followed by *C. truncatum*, *C. karstii*, *C. cliviicola*, *C. plurivorum*, and *C. boninense*. Notably, *F. acutatum*, *C. cliviicola*, *C. boninense*, and *C. fructicola* were identified for the first time as pathogens of soybean pods under the maize–soybean strip intercropping system in Southwestern China. These findings highlight emerging virulent pathogens responsible for soybean pod decay and provide a valuable foundation for understanding the pathogen population during the later growth stages of soybean.

## 1. Introduction

Soybean (*Glycine max* L.) is a globally important legume crop which has been cultivated for millennia [1,2,3]. It is a crucial source of plant-based protein (40%) and oil (20%), both essential for human nutrition and animal feed, playing a key role in global food security [4,5]. However, seed-borne diseases caused by various harmful pathogens during the reproductive stages lead to severe economic losses due to significant yield reductions, poor seed quality and compromised marketability [6]. These pathogens such as *Fusarium*, *Colletotrichum*, *Diaporthe*, *Sclerotinia*, *Cercospora*, and *Phytophthora*, have been widely reported to infect various soybean organs [7,8]. Among the most detrimental pathogens, *Fusarium* and *Colletotrichum* stand out due to their widespread prevalence and destructive impact on seed development and viability [9,10,11,12]. The genus *Fusarium* comprises ubiquitous pathogens that affect seeds, soil, and residue, are responsible for complex diseases such as root rot, pod blight, seed rot, and sudden death syndrome, all of which severely diminish germination rates and seedling vigor worldwide [13,14,15,16]. Their genetic diversity and adaptability have been well documented, with distinct species exhibiting pronounced pathogenicity across different regions. For instance, *Fusarium proliferatum* displays high aggressiveness in Hubei province, China [12], whereas *F. oxysporum*, *F. equiseti*, and *F. graminearum* are particularly pathogenic in Sichuan province [7,17,18,19,20,21]. These fungi frequently exist not only as individual species but also as pathogen complexes, complicating disease management and underscoring the urgent need for precise identification at the species level [22,23,24]. A wide spectrum of *Fusarium* species, such as *F. solani*, *F. oxysporum*, *F. acuminatum*, *F. avenaceum*, *F. cerealis*, *F. culmorum*, *F. equiseti*, *F. graminearum*, *F. proliferatum*, *F. pseudograminearum*, *F. fujikuroi*, *F. asiaticum*, *F. commune*, and *F. verticillioides*, have been associated with soybeans being isolated from various tissues [25,26,27,28,29,30,31,32]. Numerous studies investigating cultivar resistance, pathogenicity, distribution and incidence rates provide evidence that colonization of soybean roots by multiple *Fusarium* species is commonplace.

However, significant knowledge gaps persist regarding the precise roles and pathogenic dynamics of these pathogens, specifically those affecting soybean seeds and pods, and their ultimate impact on seed quality and yield [30,33]. Similarly, *Colletotrichum*, ranked among the top 10 most significant plant pathogenic fungi globally, poses a substantial threat. Its exceptionally broad host range (infecting over 3000 plant species) and capacity for latent infections make effective control exceptionally challenging [34,35,36]. In soybean, *Colletotrichum* species can induce anthracnose at all developmental stages [37], with symptoms manifesting as leaf spotting, stem lesions, pod necrosis and premature defoliation, all contributing to considerable yield losses [38]. Crucially, these pathogens are primarily seed-transmitted, and infected seeds lead to damping-off of seedlings and the development of lesions on cotyledons during the V1 and V2 developmental stages [39]. The genetic diversity within this pathogen complex, along with the key epidemiological and biological characteristics of its constituent members, remains poorly characterized, necessitating more precise and comprehensive studies [40,41,42,43,44]. For both *Fusarium* and *Colletotrichum* pathogens, accurate identification at the species level is an indispensable foundation for developing effective disease-management strategies. Moreover, traditional morphological methods often prove inadequate due to overlapping characteristics within species complexes [45,46]. Consequently, molecular approaches, particularly multi-locus phylogenetic analysis targeting conserved genes such as Internal transcribed spacer (*rDNA ITS*), Translation elongation factor gene (*EF1-α*), RNA polymerases II second largest subunit *(RPB2*) for *Fusarium* isolates, and β-tubulin (*β-tubulin*), Glyceraldehyde 3-phosphate dehydrogenase (*GADPH*), Chitin Synthase 1 (*CHS-1*), Actin (*ACT*), Beta-tubulin II (*TUB2*), and Calmodulin (*CAL*) for *Colletotrichum* isolates [47,48,49], have become the standard for robust species delineation and understanding population dynamics. Such precision is vital for tracking emerging isolates and designing targeted interventions. Intercropping systems are widely adopted due to their efficient utilization of light resources, improvement of soil structure through microbes, significant reduction in weeds and pests, and higher productivity under eco-friendly conditions compared to monoculture systems [21]. In Southwestern China, particularly Sichuan province, the widespread adoption of maize–soybean relay strip intercropping enhances land productivity (high Land Equivalent Ratio, LER > 1.5), soil health, resource-use efficiency, and disease and pest suppression [50,51]. However, the characteristic high humidity, moderate temperatures, and limited sunlight in this region create a microenvironment highly conducive to fungal proliferation, infection, and dispersal [52,53]. Studies confirm that these cool and high humid conditions significantly shape pathogenic fungal communities and intensify disease pressure, posing a persistent threat to soybean production [54]. Emerging evidence suggests that pathogen populations exhibit rapid adaptation, complex genetic diversity, and increased aggressiveness, potentially linked to changing climatic conditions and evolving agricultural practices [55]. Despite this, comprehensive data on the diversity and pathogenicity of seed- and pod- associated fungi, particularly within the distinctive intercropping pattern in Sichuan province, remains limited. Therefore, this study aims to systemically isolate and characterize the mycobiota associated with soybean seeds, focusing on *Fusarium* and *Colletotrichum* species, based on multi-locus phylogenetic analysis. Additionally, the in vitro pathogenicity of typically dominant *Fusarium* and *Colletotrichum* isolates will be evaluated. The findings of the current study offer valuable insight into the composition and threat level of major seed pathogens in a critical soybean growing region with distinctive intercropping cultivation and are expected to lay a solid scientific foundation for developing disease-management strategies and soybean resistance breeding.

## 2. Materials and Methods

### 2.1. Sampling and Fungal Isolation

A survey was conducted at experimental sites to collect soybean pods (R6 stage: Full seed) depicting discoloration, decay, and the presence of mycelium under maize–soybean strip intercropping from five different soybean-cultivating regions (Changzhou, Renshou, Nanchong, Jianyang, and Zigong) in Sichuan province, Southwestern China, in 2023. A total of 182 pods were collected and used for fungal isolation. In brief, the fungal pathogens were isolated by washing pod samples under running tap water, followed by drying, excising into small fragments (4–8 mm), and surface sterilization using 1% sodium hypochlorite (*v*/*v*) for 1 min and 75% ethanol (*v*/*v*) for 2 min. The small fragments were washed thrice, dried on sterile filter paper, and transferred to potato dextrose agar plates (PDA; potato 200 g L^−1^, glucose anhydrous 10 g L^−1^, and agar 15 g L^−1^). The plates were incubated for 7–15 days at 25 ± 2 °C in complete darkness, and the fungal isolates were purified by transferring active marginal hyphae onto fresh PDA plates [56].

### 2.2. Morphological Identification of Fungal Population

The morphological identification of fungal isolates was executed by observing colony feature and texture, the length and width of macroconidia, number of septa, conidial shape and size, and growth rate. These fungal isolates were incubated at 25 ± 2 °C and colony growth was recorded after two days of incubation, while the growth rate of *Fusarium* species were documented after 7 days of incubation in dark conditions. For morphological identification of *Colletotrichum* species, except for growth rate (two days post-inoculation), the other cultural variables were distinguished after 15 days post-incubation. In addition, the general PDA and species-specific CMC (Carboxymethyl-Cellulose 15.0 g L^−1^, KH_2_PO_4_ monobasic 1.0 g L^−1^, NH_4_NO_3_ 1.0 g L^−1^, yeast extract 1.0 g L^−1^, and MgSO_4_·7H_2_O 0.5 g L^−1^ in distilled water) media were used for the conidial spore production of *Fusarium* species. Similarly, PDA medium was utilized to generate enough spores of *Colletotrichum* species accordingly. The number and size of conidia (*n* = 50) were recorded carefully for each fungal species by observing them under the accessible compound microscope (Nikon Eclipse 80i, Sendai, Japan) [56].

### 2.3. Molecular Identification of Fungal Genera

The 7-day-old mycelium of fungal isolates cultured on PDA plates were scraped with disinfected blades to extract the genomic DNA, following the standard manual of the Rapid Fungi Genomic DNA Isolation Kit (Sangon Biotech, Shanghai, China). The quality and quantity of DNA were assessed using a NanoDrop™ 2000 Spectrophotometer (Thermo Scientific, Waltham, MA, USA) after extraction. For each fungal genus, specific primer pairs (listed in Table S1) were selected for PCR amplification. A 50 µL unit of reaction mixture was prepared, containing 2 μL of each primer, 25 μL of Taq PCR Master mix, 2 μL of DNA from each isolate, and 19 μL of sterilized water. The Peltier Thermal Cycler (S-1000TM, Bio-Bri, China) was used for amplification, and the temperature conditions for different primers are mentioned in Table S1. The amplified products were subjected to electrophoresis on a 1.0% (*w*/*v*) agarose gel in 1 × TAE buffer, and samples were sequenced using the ABI-PRISM 3730 automatic sequencer (Applied Biosystems, Foster City, CA, USA).

### 2.4. Phylogenetic Analysis

To evaluate the genetic differences among different fungal genera, molecular evolutionary and phylogenetic analysis were executed. The amplified sequences were trimmed with BioEdit software (developed by Tom Hall; BioEdit free download v.7.0.5.3) and then blasted against multiple databases, including *FUSARIUM*-ID and *Fusarium* MLST for *Fusarium* species [57], and the National Center for Biotechnology Information (NCBI) for *Colletotrichum* species. Additionally, Clustal X 1.83 was used for alignment of sequences of each constructed tree by removing gaps (missing barcode information) and weighing the characters universally. Phylogenetic trees of combined barcode for *Fusarium* and *Colletotrichum* species were constructed accordingly using MEGA version X with the Neighbor-Joining method, supported by the Tamura–Nei model, respectively [58]. The constructed tree clades were supported by 1000 bootstrap replicates and resulted sequences were deposited in the NCBI GenBank and TreeBASE (www.treebase.org accessed at 9 October 2024).

### 2.5. Pathogenicity Test of Isolated Fungi

For pathogenicity tests, seeds of the soybean cultivar ‘Nandou12’ and soybean pods were inoculated with spore suspension of each respective fungal species to fulfill Koch’s postulates, following the method described by [59] with minor modifications. Three representative isolates of each fungal species were selected randomly to analyze their pathogenic impact on seeds and pods, respectively. *Fusarium* spores were produced by adding 3–5 mycelial disks to 20 mL of PDA or CMC medium and then incubated in orbital shaker at 150 r·min^−1^ at 25 °C for 7 days. Similarly, spores of *Colletotrichum* isolates were obtained by scraping fungal mycelium in deionized water. The final spore concentration was adjusted to 1 × 10^5^ spore per mL with double-distilled water (ddH_2_O) for inoculation. The seeds and pods were subjected to surface sterilization with 1% NaClO and rinsed 3 times for 1 min, followed by air drying under sanitized conditions on double-layered filter paper [60]. Three separate isolates of each inoculated fungal species were used, each containing 15 seeds per plate and 3 pods for each isolate. The seeds and pods were dipped in spore suspension for 15 min to allow disease development [45]. Seeds treated with ddH_2_O served as the negative control and all plates were incubated in the dark at 25 ± 2 °C for 7 days with 70% relative humidity. After incubation, disease severity index (DSI) was assessed as described by [8] with minor modifications. In addition, the percentage of mycelium coverage area (PMC) and the seed and pod weights were noted. Finally, the tested *Fusarium* and *Colletotrichum* pathogens were re-isolated from the inoculated soybean seeds and pods. The DSI and PMC were calculated using the following formulae:

$$DSI=\frac{\sum \left(Severity\;rating\;\times \;Seed/pod\;number\;per\;rating\right)}{\left(Number\;of\;total\;seeds\;\times \;highest\;severity\;rating\right)}\times 100$$

$$PMC(\%)=\frac{Area\;covered\;by\;mycelium}{Number\;of\;total\;seed/pod\;surface\;area}\times 100$$

### 2.6. Data Process and Analysis

The recorded data was processed through Microsoft office excel 2016 (Microsoft Corporation, Redmond, WA, USA). The DSI and PMC average values were calculated from independent triplicates in pathogenicity tests. In addition, the seed and pod weights were recorded for each of the representative isolates of *Fusarium* and *Colletotrichum* species. The isolation frequency was calculated using the percentage of isolates of each species in the total isolates of the *Fusarium* or *Colletotrichum* genus. The statistical analysis was performed by applying Tukey’s test in IBM SPSS Statistics 20 (IBM Corp., Armonk, NY, USA) to underpin the significant differences (*p* > 0.05).

## 3. Results

### 3.1. Identification of Fungal Species Associated with Intercropped Soybean Pods

**Figure 1:**
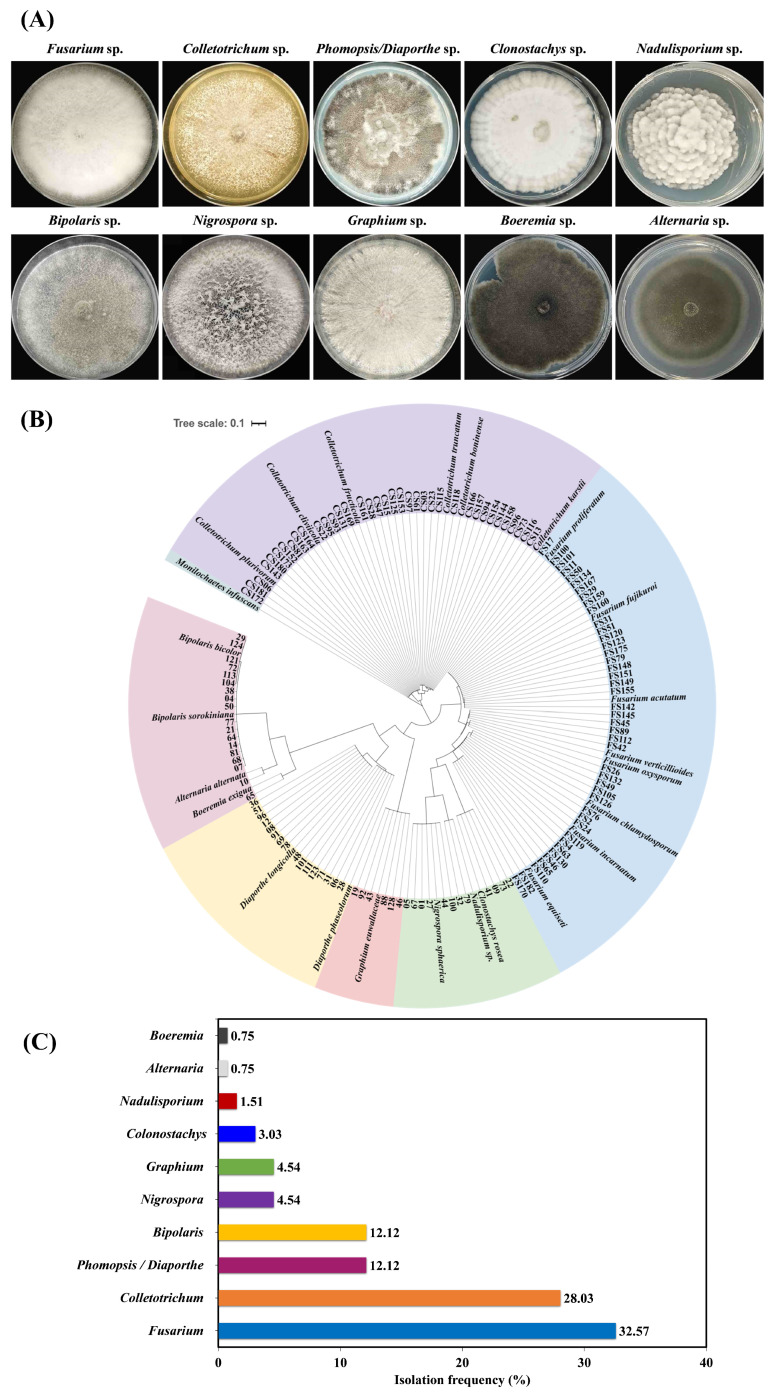
The obtained mycoflora infecting soybean pods based on morphological features and sequence analysis of *rDNA ITS* fragments. (**A**) Colonial morphology of fungal isolates, (**B**) Phylogenetic tree of fungal isolates constructed using *rDNA ITS*. (**C**) The isolation frequency of fungal isolates associated with intercropped soybean pods. The colonies were observed after 7 days of incubation on PDA. The phylogenetic tree including 132 obtained isolates and 25 reference isolates, and an outgroup *Monilochaetes infuscans* was constructed by MEGA X, with branches showing values >70 were excluded. Bootstrap support values were calculated from 1000 replications. *Monilochaetes infuscans* was used as outgroup.

In the present study, soybean pods (*n* = 182) were collected from soybean under maize–soybean strip intercropping pattern and a total of 132 isolates were obtained from infected soybean pods. Upon assessment of morphological characteristics including colonial color and the texture of colonies and mycelium, these isolates were primarily clustered into ten groups (Figure 1A). Furthermore, *rDNA ITS* fragments were amplified, sequenced, and BLASTn analysis revealed these isolate groups represented ten distinct genera, including *Fusarium*, *Colletotrichum*, *Phomopsis/Diaporthe*, *Bipolaris*, *Nigrospora*, *Graphium*, *Clonostachys*, *Nadulisporium*, *Alternaria*, and *Boeremia*. Phylogenetic analysis showed that different fungal genera were not separated clearly and clustered with other corresponding reference isolates (Figure 1B, Table S2).

To analyze the isolation frequency of these fungal genera associated with soybean pods, we found that genus *Fusarium* (32.57%) was frequently isolated from intercropped soybean pods, followed by *Colletotrichum* (28.03%), *Phomopsis/Diaporthe* (12.12%), *Bipolaris* (12.12%), *Nigrospora* (4.54%), *Graphium* (3.03%), *Clonostachys* (3.03%), and *Nadulisporium* (1.51%). Both *Alternaria* and *Boeremia* had the lowest isolation frequencies, accounting for 0.75% (Figure 1C).

### 3.2. Identification of Fusarium Species Associated with Soybean Pods

**Figure 2:**
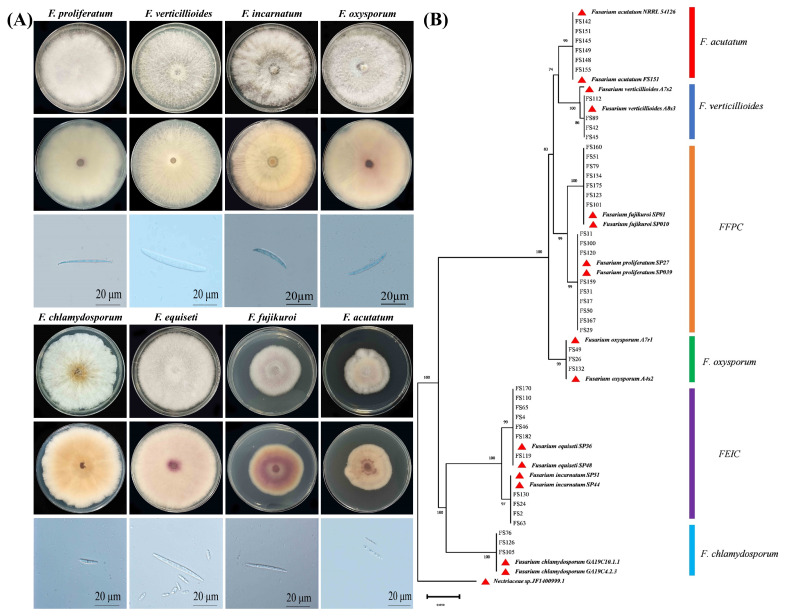
Morphological and molecular identification of *Fusarium* isolates associated with soybean pods. (**A**) Characterization of colonies and conidium of *Fusarium* isolates. Typical colonies (Top line: Colony front and Middle line: Colony back) of *Fusarium* species were observed after 7 days and macroconidia (Bottom line) were visualized under microscopy after 10 days of growth on PDA. Scale bars are 20 μm. (**B**) Phylogenetic tree of *Fusarium* isolates based on *EF1-α* and *RPB2* fragments. A maximum-likelihood (ML) tree was constructed by MEGA X (Pennsylvania State University). Bootstrap support values were ≥50% from 1000 replications, which are shown at the nodes. FFPC: *Fusarium fujikuroi*–*proliferatum* complex; FEIC: *Fusarium equiseti*–*incarnatum* complex.

To further verify *Fusarium* species, morphological features of colonies and conidial spores of different *Fusarium* isolates were observed, as shown in Figure 2A and Table 1. After seven days of incubation, *Fusarium* isolates displayed a pronounced variation in colony colors, which ranged from pale white, dark violet, and light violet to light pink. The mycelia also exhibited sparse and fluffy white mycelia, dense white or white-purple mixtures. Almost all *Fusarium* isolates produced pointed and sickle-shaped macroconidia. Based on conidial morphology, size, and colony pigmentation, a total of 43 *Fusarium* isolates clustered into eight morphological groups.

For molecular validation, the *EF1-α* and *RPB2* genes were amplified, sequenced, and blasted against the *Fusarium* MLST and *FUSARIUM*-ID databases. Sequence similarity analysis identified eight distinct *Fusarium* species: *F. verticillioides*, *F. incarnatum*, *F. equiseti*, *F. proliferatum*, *F. fujikuroi*, *F. oxysporum*, *F. chlamydosporum*, and *F. acutatum*. For phylogenetic analysis, maximum-likelihood trees were constructed using combined *EF1-α* and *RPB2* genes. The trees included 43 *Fusarium* isolates from this study, 17 reference isolates, and *Nectriaceae* sp. (JF740999.1) serving as an outgroup (Table S3). Phylogenetic analysis clearly resolved the taxonomic relationships and genetic distances among the *Fusarium* species (Figure 2B). All eight species formed distinct clades, except within two species complexes: the *F. equiseti*-*incarnatum* complex (FEIC) and the *F. fujikuroi*–*proliferatum* complex (FFPC). These species complexes grouped in the same major clade but formed well-supported distinct subclades. Bootstrap support values exceeded 92% for all species and species complex branches. The generated sequences were deposited in GenBank and accession numbers are provided in Table S4.

### 3.3. Identification of Colletotrichum Species Associated with Soybean Pods

**Figure 3:**
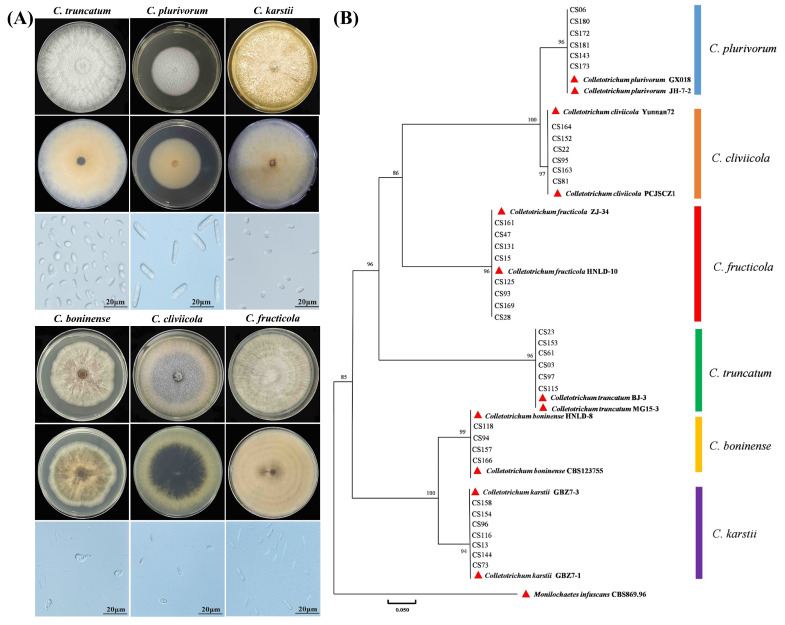
Morphological and molecular identification of *Colletotrichum* isolates associated with soybean pods. (**A**) The morphological traits of *Colletotrichum* species isolated from soybean. Colonies (Top line: Colony front, Middle line: Colony back, and Bottom line: macroconidia) of *Colletotrichum* species were examined after 7 days and spores after 15 days of incubation. Scale bars are 20 μm. (**B**) Phylogenetic tree of *Colletotrichum* isolates based on *rDNA ITS*, *CHS*, *GAPDH*, *ACT*, *CAL*, and *TUB2* genes. The tree was constructed using the ML method by MEGA X (Pennsylvania State University). Bootstrap support values were ≥50% from 1000 replications, which are shown at the nodes.

Morphological analysis of 132 fungal isolates identified 37 isolates as *Colletotrichum* species. Colonies exhibited white, cottony mycelia, while conidia varied in shape (fusiform, cylindrical and oval/ellipsoidal) presented in Figure 3A and Table 2. These isolates were classified into six morphological groups. Furthermore, *Colletotrichum* species were confirmed using a six-locus molecular approach including *rDNA ITS*, *CHS*, *GAPDH*, *ACT*, *CAL*, and *TUB2* genes. BLASTn analysis revealed maximum sequence similarity with six distinct *Colletotrichum* species. Phylogenetic analysis based on these loci employed maximum parsimony and ML methods (1000 bootstrap replicates). The tree included 37 isolates from this study, 12 reference isolates, and *Monilochaetes infuscans* (CBS:869.96) as an outgroup (Table S5). Our results demonstrated that all isolates clustered within a single major clade but resolved into six well-supported species: *C. truncatum*, *C. karstii*, *C. cliviicola*, *C. plurivorum*, *C. boninense*, and *C. fructicola*, and their accession numbers were obtained from GenBank (Figure 3B, Table S6).

### 3.4. Isolation Frequency of Fusarium and Colletotrichum Species

**Figure 4:**
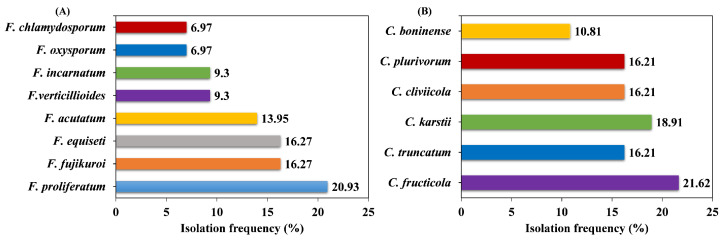
The isolation frequencies of *Fusarium* and *Colletotrichum* species from intercropped soybean pods. (**A**) *Fusarium* species and (**B**) *Colletotrichum* species.

For isolation frequency, *F. proliferatum* (20.93%), *F. fujikuroi* (16.27%), and *F. equiseti* (16.27%) were most prevalent, followed by *F. acutatum* (13.95%), *F. verticillioides* (9.3%), and *F. incarnatum* (9.3%) among the *Fusarium* genus. Compared to other species, *F. oxysporum* and *F. chlamydosporum* were the least frequent, with the isolation frequency of 6.97% each (Figure 4A). Among *Colletotrichum* species, *C. fructicola* was predominant (21.62%) followed by *C. truncatum* and *C. karstii* (18.91% each), *C. cliviicola* and *C. plurivorum* (16.21% each). *Colletotrichum boninense* was the least isolated and accounts for 10.81% of total *Colletotrichum* isolates (Figure 4B). Hence, the *F. proliferatum* and *C. fructicola* were dominant species isolated from soybean pods in Southwestern China.

### 3.5. Pathogenicity of Fusarium Species on Soybean Pods and Seeds

**Figure 5:**
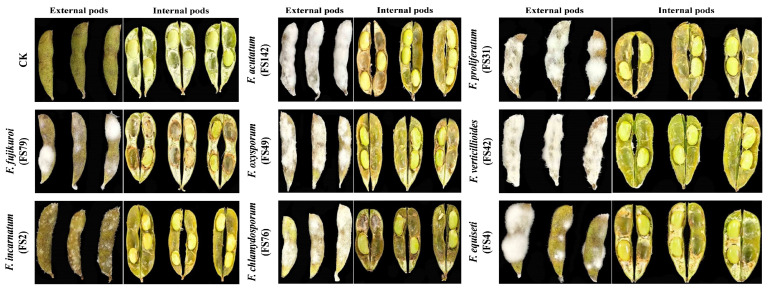
The pathogenicity of representative isolates from each *Fusarium* species on soybean pods. Soybean pods were inoculated with the selected *Fusarium* isolates by a pod-soaking inoculation method at a final concentration of 1 × 10^5^ spores per mL. The disease symptoms were observed after 7 days post-inoculation.

Pathogenicity assays were conducted to evaluate the effects of *Fusarium* species on soybean pods (Figure 5 and Table 3) and seeds (Figure S1 and Table S7). As shown in Figure 5, all species successfully penetrated soybean pods, causing varying degrees of internal seed decay. Among them, *F. acutatum* and *F. verticillioides* (100%) resulted in complete maximum PMC (100%) followed by *F. proliferatum* (90.66%), *F. equiseti* (88.33%), *F. oxysporum* (55%), *F. fujikuroi* (46.66%), *F. incarnatum* (26.66%), and *F. chlamydosporum* (23.33%). Interestingly, *F. proliferatum*, *F. acutatum*, and *F. verticillioides* exhibited a DSI of 100%, while *F. oxysporum* had a DSI of 83.33%, and *F. fujikuroi*, *F. chlamydosporum*, and *F. equiseti* all had a DSI of 75%. *Fusarium incarnatum* showed the lowest DSI (33.33%). Additionally, infected pods exhibited a reduced weight compared to un-inoculated controls, likely due to mycelial overgrowth (Table 1). Internal seed rot was observed with *F. proliferatum* and *F. fujikuroi*, while other species caused external rot with minimal discoloration compared to control pods. Seven days post-inoculation, the seeds showed partial to complete coverage by white mycelium (with noted color variation), correlating with species-specific virulence. *Fusarium acutatum* and *F. verticillioides* demonstrated maximum PMC, followed by the *F. proliferatum*, *F. oxysporum*, and *F. equiseti.* Similarly, the highest DSI was depicted by *F. acutatum* and *F. verticillioides*, followed by the *F. chlamydosporum* and *F. proliferatum*. Additionally, we re-isolated these species from the infected pods, and they exhibited the same morphological and molecular characteristics. Overall, our results demonstrate that *F. proliferatum*, *F. acutatum*, and *F. verticillioides* were the most virulent species towards soybean seeds (Figure S1, Table S7).

### 3.6. Pathogenicity of Colletotrichum Species on Soybean Pods and Seeds

**Figure 6:**
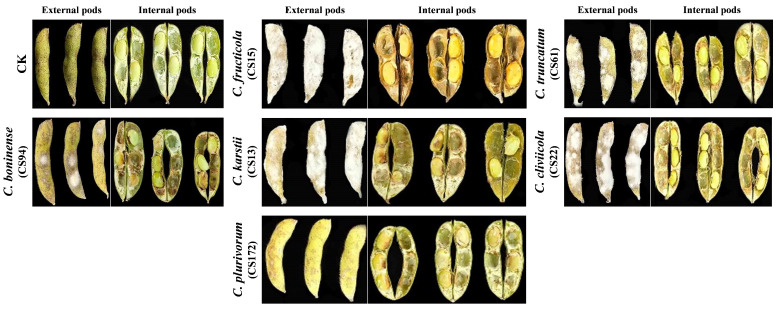
The pathogenicity of each *Colletotrichum* species representative isolates on soybean pods. Soybean pods were inoculated by the chosen *Colletotrichum* isolates by using the pod-soaking inoculation method with the final concentration of 1 × 10^5^ spores per mL. The disease symptoms were noted after 7 days post-inoculation.

All *Colletotrichum* species caused diseases on soybean pods, characterized by rotted pods, discoloration, and abundant mycelial coverage on pods (Figure 6). All representative isolates of *Colletotrichum* species resulted in 100% disease incidence on soybean pods (Table 4). *Colletotrichum karstii* (91.66%) and *C. fructicola* (90%) exhibited the highest PMC, followed by *C. truncatum* and *C. cliviicola* (both 81.66%). In contrast, *C. boninense* (11.66%) and *C. plurivorum* (10%) showed minimal PMC. DSI was highest for *C. fructicola* (100%) followed by *C. truncatum* and *C. karstii* (both 83.33%), *C. cliviicola* (66.66%), and *C. plurivorum* and *C. boninense* (both 33.33%). Similarly, pod weight varied significantly across species. *Colletotrichum boninense* recorded the highest weight (2.98 g) followed by *C. karstii* (2.67 g), *C. plurivorum* (2.59 g), *C. cliviicola* (2.21 g), *C. truncatum* (2.16 g), and *C. fructicola* (2.09 g). Furthermore, internal seed rot with discoloration occurred in pods infected by *C. fructicola*, *C. truncatum*, *C. karstii*, and *C. cliviicola*, while *C. plurivorum* and *C. boninense* caused only external discoloration. Based on disease-severity metrics and symptoms, *C. fructicola* emerged as the most virulent pathogen on soybean pods (Figure 6). Similarly, *Colletotrichum* species also caused severe damage to inoculated soybean seeds (Figure S2), with all species covering seeds with mycelium. Among these species, representative isolates of *C. fructicola* had the highest PMC (100%) followed by *C. cliviicola* (96.66%) and *C. boninense* (93.33%). However, maximum DSI occurred in *C. fructicola* and *C. truncatum* (both 91.66%), trailed by *C. boninense* (81.66%), and *C. cliviicola* (70%). Comparably, *C. karstii* and *C. plurivorum* exhibited the lowest pathogenicity, with a DSI of 58.33% and 38.33%, respectively. In contrast, *C. plurivorum* and *C. boninense* caused only external damage with slight discoloration (Table S8). Finally, we obtained the same species characterization of inoculated *Colletotrichum* isolates through re-isolation from infected pods. Thus, *C. fructicola* was the most virulent species on both soybean pods and seeds.

## 4. Discussion

It is well known that fungal diseases leading to soybean seed and pod deterioration significantly reduce global yield and quality [61]. Several *Fusarium* species, including *F. fujikuroi*, *F. graminearum*, *F. proliferatum*, and *F. equiseti–incarnatum* complex species, have been reported to infect soybean pods, with *F*. *fujikuroi* exhibiting the highest aggressiveness under maize–soybean strip intercropping in Southwestern China [8]. Additionally, *F. fujikuroi*, *F. proliferatum*, *F. verticillioides*, *F. asiaticum*, and *F. incarnatum* have also been identified as pathogens responsible for seed decay in intercropped soybean [11]. In the present study, we focused on two of the most prevalent genera, *Colletotrichum* and *Fusarium*, due to their dominant occurrence and association with soybean pod decay in Sichuan province [8,11]. Through integrated morpho-molecular characterization and multi-locus phylogenetic analysis, we identified eight *Fusarium* species, including *F. verticillioides*, *F. incarnatum*, *F. equiseti*, *F. proliferatum*, *F. fujikuroi*, *F. oxysporum*, *F. chlamydosporum*, and *F. acutatum*. Among these, *F. verticillioides*, *F. oxysporum*, *F. chlamydosporum*, and *F. acutatum* were reported for the first time as causal agents of soybean pod decay in this region, thereby extending previous findings [8]. Notably, several of these species such as *F. oxysporum*, *F. fujikuroi*, *F. verticillioides, F. proliferatum*, and the *F. incarnatum–equiseti species complex* (*FIEC*), as well as *F. chlamydosporum* have been previously implicated in soybean root rot, seed decay, and pod decay [8,11,25]. Surprisingly, *F. acutatum* was identified as the novel pathogen infecting soybean pods under maize–soybean strip intercropping in Southwestern China.

Furthermore, species within the genus *Colletotrichum* ranked as the eighth most devastating and wide-spectrum plant pathogen globally [35], are known to cause anthracnose in soybean and related legumes [62]. This genus has also been reported as the second most abundant genus associated with soybean seed decay in Southwestern China [11]. In this study, we identified six *Colletotrichum* species, including *C. truncatum*, *C. karstii*, *C. cliviicola*, *C. plurivorum*, *C. boninense*, and *C. fructicola*. Among them, *C. truncatum* has been documented as a causal agent of soybean pod blight in Chhattisgarh (India) [63] and Brazil [64], while *C. plurivorum*, a recently classified species, has been associated with soybean disease [65]. Similarly, *C*. *fructicola* and *C*. *karstii* have been reported to cause anthracnose on soybean leaves and pods, leading to yield loss [66]. Importantly, *C. cliviicola*, *C. boninense*, and *C. fructicola* were newly identified as pathogens of soybean pods in this region. Accurate pathogen identification is vital for effective disease management [40]. Advances in fungal disease identification have significantly improved the ability to identify a wide range of pathogenic plant fungi by employing specific gene sequence analysis and improved molecular techniques [67,68]. It is well established that both *Fusarium* and *Colletotrichum* species often appear in complexes, sharing similar morphological characteristics (e.g., colony and spore shape) [69]. To characterize these species, the amplification of two or more genes has emerged as a standard method for accurately identifying specific fungal species within widely spread genera [70]. Through analysis of morphological features, molecular procedures, and phylogenetic analysis of *RPB2* and *EF1-α* gene sequences, we identified 43 different *Fusarium* species. It is assumed that two sequencing sections, *EF1-α* and RNA polymerase largest subunit *RPB1* and/or *RPB2* are indispensable for *Fusarium* species characterization [71]. Numerous recent studies have used these regions for the precise identification of *Fusarium* isolates complexes [9,23,24,72]. For *Colletotrichum* species identification, we used multi-locus analysis of *ACT*, *CHS*, *ITS*, *GAPDH*, *TUB2*, and *CAL* genes. Employing these genes, we identified six different *Colletotrichum* species, including *C. boninense*, *C. truncatum*, *C. cliviicola*, *C. karstii*, *C. fructicola*, and *C. plurivorum*. A similar method has been used to identify *Colletotrichum* isolates infecting olive trees and Tea-Oil Camellia (*Camellia oleifera* C. Abel) [25,26].

Pathogenicity evaluations revealed tissue-specific virulence. Among *Fusarium* isolates, *F. acutatum* and *F. verticillioides* exhibited the highest aggressiveness (PMC and DSI) on soybean seeds. Earlier studies have shown that *F. verticillioides* can significantly diminish soybean seed quality [52]. Among the representative isolates, *C. truncatum* and *C. fructicola* exhibited the greatest virulence when inoculated on soybean seeds. Interestingly, several past studies have identified *C. fructicola* as a nonhost specific pathogen capable of infecting a number of plants and crops, including blueberry [22], sugarcane [29], and apple [73]. Besides *C. truncatum* and *C. fructicola*, our results showed that *C. karstii* acted as a moderately aggressive pathogen toward soybean pods and seeds. Consistent with our findings, *C. karstii* has been reported to cause anthracnose in soybean in China [66]. Many studies have documented that soybean pod diseases lead to seed deterioration, negatively affecting seed germination and reducing overall yield in soybean fields [74]. The pathogenicity results from our study confirmed that *Fusarium* and *Colletotrichum* isolates are capable of decaying soybean pods and seeds, as indicated by reduced seed weight and increased PMC and DSI, which could directly impact soybean yield. We predict that *F. acutatum*, *F. verticillioides*, *C. fructicola*, and *C. truncatum* are destructive plant pathogens responsible for soybean pod and seed decay. Therefore, understanding their infection mechanisms through molecular analysis provides critical insights for developing targeted management strategies.

## 5. Conclusions

This study underscores the significance of pathogen identification and management in reducing yield losses in soybean production. By isolating pathogens from soybean pods across five different locations in Sichuan Province, Southwestern China, we found that *Colletotrichum* and *Fusarium* were the most predominant genera. Based on both morphological and molecular characteristics, we identified eight *Fusarium* species and six *Colletotrichum* species. Pathogenicity tests revealed that *F. verticillioides*, *F. acutatum*, and *F. proliferatum* in the *Fusarium* genus, as well as *C. fructicola* and *C. truncatum* in the *Colletotrichum* genus, were the most aggressive. Notably, *F. acutatum*, *C. cliviicola*, *C. boninense*, and *C. fructicola* were identified for the first time as pathogens of soybean pods under the maize–soybean strip intercropping system in Southwestern China. These findings highlight the serious threat posed by diverse *Fusarium* and *Colletotrichum* species to soybean production in this region. Specifically, the environmental conditions characterized by high humidity, frequent rainfall, and relatively low temperatures during pod formation and preharvest stages create a conducive environment for fungal infection and spread. Therefore, this study provides a critical basis for better understanding the pathogen population associated with soybean pods during later growth stages of soybean under maize–soybean relay strip intercropping.

## Figures and Tables

**Table 1:** Morphological characters of *Fusarium* species cultured on PDA medium.

Species	Macroconidia				Colony Characterization	Growth Rate (cm/day)
	Shape	Width (μm)	Length (μm)	Septa		
*F. equiseti*	Falcate	3.10 ± 0.02 c, 3.02–3.70	39.25 ± 1.81 a, 38.23–45.98	3–5	Pale gray color (front), ginger yellowish (back)	4.88 ± 0.41 b
*F. incarnatum*	Falcate	3.98 ± 0.44 a, 5.67–2.72	36.98 ± 3.63 a, 45.55–36.62	3–4	Pale gray color (front), yellowish color (back)	5.32 ± 0.39 a
*F. verticillioides*	Fusiform	3.40 ± 0.90 b, 3.80–3.33	23.25 ± 0.2 b, 20.21–25.90	2–3	Pale gray colonies, reverse pale gray	4.90 ± 0.30 b
*F. proliferatum*	Falcate, fusiform	3.60 ± 1.12 b, 5.41–2.96	39.12 ± 6.54 a, 48.56–32.66	3–4	Pale gray color (front), pale gray (back)	4.50 ± 0.03 c
*F. fujikuroi*	Falcate	2.42 ± 0.46 e, 3.12–2.28	39.92 ± 1.98 a, 43.82–38.94	3–5	Pale gray color (front), pale yellowish color (back)	4.76 ± 0.32 c
*F. oxysporum*	Falcate	3.10 ± 0.16 c, 2.31–4.82	26.90 ± 1.60 b, 28.62–22.23	3	Pale gray (front) pale purple on the back	5.40 ± 0.30 a
*F. chlamydosporum*	Falcate	3.20 ± 0.82 c, 3.90–3.22	25.45 ± 0.20 b, 28.12–23.95	2–3	Brown, light pink (Front) purple (back)	5.23 ± 0.01 a
*F. acutatum*	Falcate	2.79 ± 1.62 d, 3.20–1.98	23.24 ± 0.8 b, 24.56–20.86	3–5	White-gray (front) purple (back)	4.39 ± 0.02 c

Notes: All data represent the average values from three independent replicates of each *Fusarium* species. Different lowercase letters within the same column indicate a significant variation, as determined by performingTukey’s test at a significance level of *p* > 0.05.

**Table 2:** Morphological characterization of *Colletotrichum* species cultured on PDA medium.

Species	Conidial Shape	Conidia Size		Texture	Growth Rate (cm/day)
		Length (μm)	Width (μm)		
*C. truncatum*	Fusiform	23.20 ± 0.56 a 24.65–16.22	5.56 ± 0.35 b 5.90–4.32	Cottony	6.90 ± 0.12 a
*C. karstii*	Cylindrical	15.5 ± 0.20 b 18.20–14.90	6.80 ± 0.23 a 8.56–5.52	Cottony	5.63 ± 0.27 b
*C. cliviicola*	oval/ellipsoidal	13.35 ± 0.02 c 14.22–12.86	3.62 ± 0.06 c 4.56–3.22	Cottony	6.12 ± 0.25 a
*C. plurivorum*	Fusiform	13.75 ± 0.12 c 15.78–12.66	3.4 ± 0.02 c 4.45–3.56	Cottony and white	6.30 ± 0.09 a
*C. boninense*	Cylindrical	15.10 ± 0.20 b 16.20–14.75	5.30 ± 0.45 b 6.60–4.25	Medium brown	6.15 ± 0.02 a
*C*. *fructicola*	Fusiform	12.90 ± 0.32 c 14.56–10.86	6.80 ± 0.23 a 8.56–5.52	Grayish black	6.4 ± 0.60 a

Notes: All data are the means of three independent replicates of each *Colletotrichum* test. Different lowercase letters in the same column reveal a significant variation after Tukey’s test analysis at the level of *p* > 0.05, *n* = 50.

**Table 3:** Pathogenicity of representative isolates from each *Fusarium* species on soybean pods isolated from intercropped soybean pods.

Isolates	PMC (%)	DSI (%)	Pod Weight (g)
Control (CK)	0 ± 0 e	0 ± 0 d	1.89 ± 0.04 d
*F. proliferatum* (FS31)	90.66 ± 0.40 a	96.66 ± 4.71 a	1.79 ± 0.31 c
*F. proliferatum* (FS120)	89.66 ± 0.47 a	100 ± 0 a	1.71 ± 0.05 c
*F. proliferatum* (FS167)	78.33 ± 2.35 a	93.33 ± 4.71 a	1.74 ± 0.01 c
*F. fujikuroi* (FS79)	43.33 ± 2.35 b	66.66 ± 11.78 b	2.48 ± 0.27 b
*F. fujikuroi* (FS101)	46.66 ± 4.71 b	75 ± 0 ab	2.37 ± 0.24 b
*F. fujikuroi* (FS123)	23.33 ± 2.35 c	58.33 ± 11.78 b	2.71 ± 0.09 b
*F. equiseti* (FS4)	88.33 ± 2.35 a	75 ± 11.78 b	2.21 ± 0.07 b
*F. equiseti* (FS65)	41.66 ± 2.35 b	50 ± 11.78 b	2.19 ± 0.02 b
*F. equiseti* (FS170)	21.66 ± 2.35 c	50 ± 20.41 b	2.13 ± 0.03 b
*F. acutatum* (FS142)	100 ± 0 a	100 ± 0 a	2.64 ± 0.09 b
*F. acutatum* (FS151)	95 ± 4.08 a	100 ± 0 a	2.47 ± 0.31 b
*F. acutatum* (FS155)	98.33 ± 2.35 a	100 ± 0 a	2.57 ± 0.12 b
*F. verticillioides* (FS42)	98.33 ± 2.35 a	100 ± 0 a	2.29 ± 0.12 b
*F. verticillioides* (FS89)	100 ± 0 a	100 ± 0 a	2.25 ± 0.04 b
*F. verticillioides* (FS112)	100 ± 0 a	100 ± 0 a	2.29 ± 0.06 c
*F. incarnatum* (FS2)	26.66 ± 2.35 c	33.33 ± 11.78 c	3.34 ± 0.19 a
*F. incarnatum* (FS24)	20 ± 4.08 dc	25 ± 0 c	3.29 ± 0.05 a
*F. incarnatum* (FS130)	18.33 ± 6.23 d	33.33 ± 11.78 c	3.25 ± 0.06 a
*F. oxysporum* (FS49)	55 ± 4.08 b	83.33 ± 11.78 a	1.84 ± 0.27 c
*F. oxysporum* (FS26)	45 ± 4.08 b	66.66 ± 11.78 b	1.86 ± 0.04 c
*F. oxysporum* (FS132)	50 ± 4.08 b	58.33 ± 11.78 b	1.89 ± 0.16 c
*F. chlamydosporum* (FS76)	18.33 ± 2.35 d	75 ± 0 ab	1.68 ± 0.16 c
*F. chlamydosporum* (FS105)	23.33 ± 6.23 c	50 ± 20.41 b	1.57 ± 0.05 c
*F. chlamydosporum* (FS126)	16.66 ± 2.35 d	66.66 ± 11.78 b	1.69 ± 0.08 c

Notes: The data are the average values from three independent replicates of each *Fusarium* species. Lowercase letters in the same column indicate significant difference. Significant difference was analyzed using Tukey’s test at the level of *p* > 0.05. The different lowercase letters highlight the significant differences of each parameter within *Fusarium* species.

**Table 4:** Pod pathogenicity of representative isolates of each *Colletotrichum* species obtained from intercropped soybean pods.

Isolates	PMC (%)	DSI (%)	Pod Weight (g)
Control (CK)	0 ± 0 d	0 ± 0 e	1.86 ± 0.040 d
*C. fructicola* (CS15)	90 ± 4.08 a	100 ± 0 a	2.51 ± 0.04 b
*C. fructicola* (CS93)	86.66 ± 6.23 a	91.66 ± 11.78 a	2.52 ± 0.02 b
*C. fructicola* (CS169)	76.66 ± 2.35 ab	100 ± 0 a	2.51 ± 0.01 b
*C. truncatum* (CS03)	78.33 ± 4.71 ab	75 ± 0 b	2.09 ± 0.02 c
*C. truncatum* (CS61)	80 ± 4.08 a	83.33 ± 11.78 b	2.11 ± 0.01 c
*C. truncatum* (CS153)	81.66 ± 2.35 a	66.66 ± 11.7 b	2.16 ± 0.08 c
*C. karstii* (CS13)	91.66 ± 2.35 a	83.33 ± 11.78 b	2.67 ± 0.05 b
*C. karstii* (CS96)	86.66 ± 2.35 a	75 ± 0 b	2.47 ± 0.08 b
*C. karstii* (CS158)	80 ± 7.07 a	75 ± 0 b	2.67 ± 0.03 b
*C. cliviicola* (CS22)	81.66 ± 2.35 a	66.66 ± 11.78 c	2.13 ± 0.12 c
*C. cliviicola* (CS95)	76.66 ± 2.35 ab	58.33 ± 11.78 c	2.21 ± 0.08 c
*C. cliviicola* (CS164)	65 ± 4.0 b	50 ± 20.41 c	2.26 ± 0.09 c
*C. plurivorum* (CS06)	10 ± 4.08 c	25 ± 0 d	2.60 ± 0.02 b
*C. plurivorum* (CS172)	8.33 ± 2.35 c	33.33 ± 11.78 d	2.59 ± 0.01 b
*C. plurivorum* (CS180)	6.66 ± 2.35 c	25 ± 20.41 d	2.61 ± 0.01 b
*C. boninense* (CS94)	10 ± 4.08 c	33.33 ± 11.78 d	2.96 ± 0.01 a
*C. boninense* (CS118)	11.66 ± 6.23 c	25 ± 0 d	2.98 ± 0.02 a
*C. boninense* (CS166)	10 ± 4.08 c	25 ± 0 d	2.95 ± 0.03 a

Notes: The data are the mean values from three independent replicates of each *Colletotrichum* species. Lowercase letters in the same column indicate significant difference. Significant difference was calculated by applying Tukey’s test at the level of *p* > 0.05. The different lowercase letters represent the significant differences of each parameter among *Colletotrichum* species.

## Data Availability

The gene sequence information of *Fusarium* and *Colletotrichum* isolates in this study are available in the database of NCBI.

## Supplementary Materials

The following supporting information can be downloaded at: https://www.mdpi.com/article/10.3390/pathogens14101020/s1. Table S1: The primers used for amplification and identification of *Fusarium* and *Colletotrichum* species. Table S2: Reference sequences of *rDNA ITS* gene from NCBI used for the homology analysis of isolated fungal diversity. Table S3: Reference sequences of *RPB2* and of *EF1-α* gene from Fusarium MLST, GenBank, and FUSARIUM-ID used for the homology analysis of isolated *Fusarium* species. Table S4: The GenBank accession numbers of *RPB2* and *EF1-α* of *Fusarium* species obtained from soybean pods. Table S5: Reference gene sequences from NCBI GenBank used for the homology analysis of *Colletotrichum* species. Table S6: The gene bank accession numbers of *CHS*, *ITS*, *GADPH*, *ACT*, *CAL*, and *TUB2* gene from NCBI. Table S7: Seed pathogenicity of *Fusarium* species isolated from intercropped soybean pods. Table S8: Seeds pathogenicity of *Colletotrichum* species isolated from intercropped soybean pods. Figure S1: Seed symptoms after inoculation with the representative isolates of different *Fusarium* species from intercropped soybean pods. Figure S2: Seed symptoms after inoculation with the representative isolates of different *Colletotrichum* species from intercropped soybean pods.
