# Supplementary material for: Morpho-Molecular Identification and Pathogenic Characterization of Fusarium and Colletotrichum Species Associated with Intercropped Soybean Pod Decay
Source: Pathogens. 2025 Oct 8;14(10):1020. doi: 10.3390/pathogens14101020 (PMC12567077; doi:10.3390/pathogens14101020)
Supplement: Supplementary file 1 [file pathogens-14-01020-s001.zip › pathogens-3843172-supplementary.pdf]

pathogens

MDPI

Article

Morpho-Molecular Identification and Pathogenic Characterization of *Fusarium* and *Colletotrichum* Species Associated with Intercropped Soybean Pod Decay

Maira Munir [1], Muhammd Naeem [2], Xiaoling Wu [1], Weiying Zeng [3], Zudong Sun [3], Yuze Li [1], Taiwen Yong [1], Feng Yang [1] and Xiaoli Chang [1,*]

1 College of Agronomy, Sichuan Agricultural University, Chengdu 611130, China; mairamunir23@gmail.com (M.M.); wuxl2014@163.com (X.W.); l_yz_deu@163.com (Y.L.); yongtaiwen@sicau.edu.cn (T.Y.); f.yang@sicau.edu.cn (F.Y.)
2 Institute of Crop Science, College of Agriculture and Biotechnology, Zhejiang University, Hangzhou 310058, China; naeem.muhammd@zju.edu.cn (M.N).
3 Institute of Economic Crops, Guangxi Academy of Agricultural Science, Nanning 530007, China; zengweiying_1981@163.com (W.Z.); zudongsun639@163.com (Z.S.)
* Correspondence: xl_chang14042@sicau.edu.cn (X.C)

Table S1: The primers used for amplification and identification of *Fusarium* and *Colletotrichum* species.

Gene	Primer name	Primer sequence (5'-3')	Annealing temperature (°C)
EF1-α	EF1-728F	CATCGAGAAGTTCGAGAAGG	55°C
	EF1-986R	TACTTGAAGGAACCCTACC	
RPB2	RPB2-7cr	CCCATRGCTTGYTTRCCCAT	58°C
	RPB2-5f2	GGGGWGAYCAGAAGAAGGC	
GADPH	GDF	GCCGTC AACGATTTGA	61°C
	GDR	GGG TGGAGTCGT ACTTGA GCATGI	
rDNA ITS	ITS1	TCCGTAGGTGAACCTGCGG	55°C
	ITS4	GCTGCGTTCTTCATCGATGC	
CHS-1	CHS-79F	TGG GGCAAG GATGCT TGGAAGAAG	55°C
	CHS-345R	TGG AAG AACCAT CTG TGAGAG TTG	
CAL	CLI	GARTWCAAGGAGGCCTTCTC	52°C
	CL2	TTT TTGCATCAT GAG TTGGAC	
ACT	ACT512F	ATGTGCAAGGCC GGTTTCGC	61°C
	ACT-783R	TACGAGTCCTTCTGGCCCAT	
TUB2	TUB2-1	AACATG CGTGAG ATTGTAAGT	61°C
	TUB2-2	TAGTGACCC TTGGCCCAGTTG	

Notes**:** Different genes were amplified at different temperature to obtained specific genetic fragment.

 https://doi.org/10.3390/pathogens14101020

Table S2: Reference sequences of *rDNA ITS* gene from NCBI GenBank used for the homology analysis of isolated fungal diversity.

**Species name**	**Isolate name**	**GenBank accession numbers**
Fusarium acutatum	RI13	PQ560110.1
Fusarium verticillioides	LAPEMI	KR052812.1
Fusarium fujikuroi	HLW-4	PV565693.1
Fusarium proliferatum	CBS:240.64	MH858428.1
Fusarium oxysporum	TOFOL-IHBT	HM484352.1
Fusarium equiseti	F.equ.ARF-1	MZ496590.1
Fusarium incarnatum	FoLC10	PQ564679.1
Fusarium chlamydosporum	Fs3	ON624336.1
Colletotrichum truncatum	MG15-3	MT982175.1
Colletotrichum cliviicola	PCJSCZ1	ON793109.1
Colletotrichum karstii	GBZ7-3	MZ962374.
Colletotrichum plurivorum	GX018	MN092338.1
Colletotrichum fructicola	ZJ-34	MK629870.1
Colletotrichum boninense	CBS	JQ005153.1
Diaporthe longicolla	ATCC 60325	NR_144924.1
Diaporthe phaseolorum	OL_MDA20	JX944176.1
Bipolaris sorokiniana	B_Bs01	PV467704.1
Bipolaris bicolor	PQRB202207	OQ080002.1
Nigrospora sphaerica	LSGDU3	PV107334.1
Clonostachys rosea	breed Clonostachys	OL691915.1
Nadulisporium sp.	PDL-005	KX708704.1
Alternaria alternata	CRL17	PP555952.1
Boeremia exigua	Pee_1	KT211361.1
Graphium euwallaceae	UCR2308	KM592371.1
Monilochaetes infuscans	CBS:869.96	JQ005780.1

Notes: The reference isolated were obtained from GenBank to construct phylogenetic tree.

Table S3: Reference sequences of *RPB2* and of *EF1-α* genes from Fusarium MLST, GenBank and FUSARIUM-ID used for the homology analysis of isolated *Fusarium* species.

***Fusarium* isolates**	**GenBank accession numbers**	
	EF1-α	***RPB2***
Fusarium fujikuroi SP01	MK611857	MN625641
Fusarium fujikuroi SP010	MK611871	MN62566
Fusarium proliferatum SP27	MK611892	MN625690
Fusarium proliferatum SP039	MK611891	MN625689
Fusarium equiseti SP36	MK611914	MN625629
Fusarium incarnatum SP44	MK611902	MN625700
Fusarium incarnatum SP51	MK611903	MN625701
Fusarium verticillioides A7s2	MK560261	MN892281
Fusarium verticillioides A8s3	MK560263	MN892279

Fusarium oxysporum A4s2	MK560300	MN892307
Fusarium oxysporum A7r1	MK560302	MN892305
Fusarium acutatum NRRL 54126	HM347129	HM347214
Fusarium acutatum FS151	PQ450963	PQ451014
Fusarium chlamydosporum GA19C10.1.1	OR441011	OR425371
Fusarium chlamydosporum GA19C4.2.3	OR441009	OR425369
Nectriaceae sp. NRRL 52754	JF740999.1	-

Notes: The reference isolates of *Fusarium* species and *Nectriaceae* sp. (Outgroup) were used to construct the phylogenetic tree. "-" means no corresponding gene sequence obtained from NCBI database.

Table S4: The GenBank accession numbers of *RPB2* and *EF1-α* genes of *Fusarium* species obtained from soybean pods.

Isolates	Gene bank accession numbers		*Fusarium species*
	RPB2	*EF1-α*	
FS1	PQ451044	PQ450993	*F. proliferatum*
FS2	PQ451040	PQ450989	*F. incarnatum*
FS4	PQ451026	PQ450975	*F. equiseti*
FS7	PQ451045	PQ450994	*F. proliferatum*
FS11	PQ451046	PQ450995	*F. proliferatum*
FS17	PQ451047	PQ450996	*F. proliferatum*
FS21	PQ451016	PQ450965	*F. fujikuroi*
FS24	PQ451041	PQ450990	*F. incarnatum*
FS26	PQ451033	PQ450982	*F. oxysporum*
FS29	PQ451048	PQ450997	*F. proliferatum*
FS31	PQ451049	PQ450998	*F. proliferatum*
FS42	PQ451036	PQ450985	*F. verticillioides*
FS45	PQ451037	PQ450986	*F. verticillioides*
FS46	PQ451027	PQ450976	*F. equiseti*
FS49	PQ451034	PQ450983	*F. oxysporum*
FS50	PQ451050	PQ450999	*F. proliferatum*
FS51	PQ451017	PQ450966	*F. fujikuroi*
FS62	PQ451051	PQ451000	*F. proliferatum*
FS63	PQ451042	PQ450991	*F. incarnatum*
FS65	PQ451028	PQ450977	*F. equiseti*
FS76	PQ451023	PQ450972	*F. chlamydosporum*
FS79	PQ451018	PQ450967	*F. fujikuroi*
FS80	PQ451052	PQ451001	*F. proliferatum*
FS89	PQ451038	PQ450987	*F. verticillioides*
FS100	PQ451053	PQ451002	*F. proliferatum*
FS101	PQ451019	PQ450968	*F. fujikuroi*
FS105	PQ451024	PQ450973	*F. chlamydosporum*
FS110	PQ451029	PQ450978	*F. equiseti*
FS112	PQ451039	PQ450988	*F. verticillioides*
FS119	PQ451030	PQ450979	*F. equiseti*
FS120	PQ451054	PQ451003	*F. proliferatum*

FS123	PQ451020	PQ450969	*F. fujikuroi*
FS124	PQ451055	PQ451004	*F. proliferatum*
FS126	PQ451025	PQ450974	*F. chlamydosporum*
FS130	PQ451043	PQ450992	*F. incarnatum*
FS132	PQ451035	PQ450984	*F. oxysporum*
FS134	PQ451021	PQ450970	*F. fujikuroi*
FS140	PQ451056	PQ451005	*F. proliferatum*
FS142	PQ451010	PQ450959	*F. acutatum*
FS145	PQ451011	PQ450960	*F. acutatum*
FS148	PQ451012	PQ450961	*F. acutatum*
FS149	PQ451013	PQ450962	*F. acutatum*
FS151	PQ451014	PQ450963	*F. acutatum*
FS155	PQ451015	PQ450964	*F. acutatum*
FS159	PQ451057	PQ451006	*F. proliferatum*
FS160	PQ451022	PQ450971	*F. fujikuroi*
FS167	PQ451058	PQ451007	*F. proliferatum*
FS168	PQ451059	PQ451008	*F. proliferatum*
FS170	PQ451031	PQ450980	*F. equiseti*
FS175	PQ451060	PQ451009	*F. proliferatum*
FS182	PQ451032	PQ450981	*F. equiseti*

***Notes*: The *Fusarium* isolates were characterized employing the homology analysis of partial sequences of *EF1-α* and *RPB2* genes on FUSARIUM-ID and n Fusarium MLST databases.

Table S5: Reference sequences of *rDNA ITS*, *CHS*, *GADPH*, *ACT*, *CAL* and of *TUB2* genes from NCBI GenBank used for the homology analysis of isolated *Colletotrichum* species.

Colletotrichum **isolates**	**GenBank accession numbers**					
	rDNA ITS	***CHS***	***GADPH***	***ACT***	***CAL***	***TUB2***
C. truncatum MG15-3	MT982175.1	MT988553.1	MT988556.1	MT988547.1	MT988550.1	MT988559.1
C. truncatum BJ-3	MK629874.1	MK675282.1	MK675260.1	MK675238.1	MK681375.1	MK681418.1
C. cliviicola PCJSCZ1	ON793109.1	OP750722.1	OP781546.1	OP750699.1	OP750986.1	OP750875.1
C. cliviicola Yunnan72	MZ165574.1	MZ352078.1	MZ361141.1	MZ277672.1	MZ361251.1	MZ277739.1
C. karstii GBZ7-3	MZ962374.1	OK040211.1	OK040216.1	OK040201.1	OK040206.1	OK040221.1
C. karstii GBZ7-1	MZ962373.1	OK040210.1	OK040215.1	OK040200.1	OK040205.1	OK040220.1
C. plurivorum GX018	MN092338	MN092331.1	MN092335.1	MN092324.1	MN092329.1	MN092341.1
C. plurivorum JH-7-2	MW507147.1	MW507370.1	MW507371.1	MW524860.1	MW507369.1	MW507372.1
C. fructicola ZJ-34	MK629870.1	MK675278.1	MK675256.1	MK675234.1	MK681371.1	MK681414.1
C. fructicola HNLD-10	MK629873.1	MK675281.1	MK675259.1	MK675237.1	MK681374.1	MK681417.1
C. boninense CBS 123755	JQ005153.1	JQ005327.1	JQ005240.1	JQ005501.1	JQ005674.1	JQ005588.1
C. boninense HNLD-8	MK629872.1	MK675280.1	MK675258.1	MK675236.1	MK681373.1	MK681416.1
Monilochaetes infuscans CBS:869.96	JQ005780	-	-	JQ005843	-	JQ005864

Notes**:** All reference isolates of *Colletotrichum* species and *Monilochaetes infuscans* (Outgroup) were used to construct the phylogenetic tree. “-” means no corresponding gene sequence obtained from NCBI database.

Table S6: The GenBank accession numbers of *CHS*, *ITS*, *GADPH*, *ACT*, *CAL* and *TUB2* genes from NCBI.

Isolates	GenBank accession number						Species name
	CHS	***ITS***	***GADPH***	***ACT***	***CAL***	***TUB2***	
CS03	PQ450938	PV688511	PQ450945	PQ450924	PQ450931	PQ450952	*C. truncatum*
CS06	PQ450906	PV688499	PQ450912	PQ450894	PQ450900	PQ450918	*C. plurivorum*
CS13	PQ450873	PV688476	PQ450880	PQ450859	PQ450866	PQ450887	*C. karstii*
CS15	PQ474698	PV688486	PQ474706	PQ474682	PQ474690	PQ474714	*C. fructicola*
CS22	PQ439807	PV710382	PQ439813	PQ439819	PQ439825	PQ439801	*C. cliviicola*
CS23	PQ450939	PV688512	PQ450946	PQ450925	PQ450932	PQ450953	*C. truncatum*
CS28	PQ474699	PV688487	PQ474707	PQ474683	PQ474691	PQ474715	*C. fructicola*
CS47	PQ474700	PV688488	PQ474708	PQ474684	PQ474692	PQ474716	*C. fructicola*
CS57	PQ450940	PV688513	PQ450947	PQ450926	PQ450933	PQ450954	*C. truncatum*
CS61	PQ450941	PV688514	PQ450948	PQ450927	PQ450934	PQ450955	*C. truncatum*
CS73	PQ450874	PV688477	PQ450881	PQ450860	PQ450867	PQ450888	*C. karstii*
CS81	PQ439808	PV710383	PQ439814	PQ439820	PQ439826	PQ439802	*C. cliviicola*
CS93	PQ474701	PV688489	PQ474709	PQ474685	PQ474693	PQ474717	*C. fructicola*
CS94	PQ562883	PV688495	PQ562887	PQ562875	PQ562879	PV232705	*boninense*
CS95	PQ439809	PV710384	PQ439815	PQ439821	PQ439827	PQ439803	*C. cliviicola*
CS96	PQ450875	PV688478	PQ450882	PQ450861	PQ450868	PQ450889	*C. karstii*
CS97	PQ450942	PV688515	PQ450949	PQ450928	PQ450935	PQ450956	*C. truncatum*
CS115	PQ450943	PV688516	PQ450950	PQ450929	PQ450936	PQ450957	*C. truncatum*
CS116	PQ450876	PV688479	PQ450883	PQ450862	PQ450869	PQ450890	*C. karstii*
CS118	PQ562884	PV688496	PQ562888	PQ562876	PQ562880	PV232706	*C. boninense*
CS125	PQ474702	PV688490	PQ474710	PQ474686	PQ474694	PQ474718	*C. fructicola*
CS131	PQ474703	PV688491	PQ474711	PQ474687	PQ474695	PQ474719	*C. fructicola*
CS143	PQ450907	PV688500	PQ450913	PQ450895	PQ450901	PQ450919	*C. plurivorum*
CS144	PQ450877	PV688480	PQ450884	PQ450863	PQ450870	PQ450891	*C. karstii*
CS152	PQ439810	PV710385	PQ439816	PQ439822	PQ439828	PQ439804	*C. cliviicola*
CS153	PQ450944	PV688517	PQ450951	PQ450930	PQ450937	PQ450958	*C. truncatum*
CS154	PQ450878	PV688481	PQ450885	PQ450864	PQ450871	PQ450892	*C. karstii*
CS157	PQ562885	PV688497	PQ562889	PQ562877	PQ562881	PV232707	*C. boninense*
CS158	PQ450879	PV688482	PQ450886	PQ450865	PQ450872	PQ450893	*C. karstii*
CS161	PQ474704	PV688492	PQ474712	PQ474688	PQ474696	PQ474720	*C. fructicola*
CS163	PQ439811	PV710386	PQ439817	PQ439823	PQ439829	PQ439805	*C. cliviicola*
CS164	PQ439812	PV710387	PQ439818	PQ439824	PQ439830	PQ439806	*C. cliviicola*
CS166	PQ562886	PV688498	PQ562890	PQ562878	PQ562882	PV232708	*C. boninense*
CS169	PQ474705	PV688493	PQ474713	PQ474689	PQ474697	PQ474721	*C. fructicola*
CS172	PQ450908	PV688501	PQ450914	PQ450896	PQ450902	PQ450920	*C. plurivorum*
CS173	PQ450909	PV688502	PQ450915	PQ450897	PQ450903	PQ450921	*C. plurivorum*
CS180	PQ450910	PV688503	PQ450916	PQ450898	PQ450904	PQ450922	*C. plurivorum*
CS181	PQ450911	PV688504	PQ450917	PQ450899	PQ450905	PQ450923	*C. plurivorum*

Notes: The *Colletotrichum* species were recognized by homology analysis *CHS*, *ITS*, *GADPH*, *ACT*, *CAL* and *TUB2* genes on GenBank database.

Table S7: Seed pathogenicity of *Fusarium* species isolated from intercropped soybean pods.

Isolates	PMC (%)	DSI (%)	Seed weight (g)	Seed symptoms
Control (CK)	0±0f	0±0e	0.316±0.01c	Healthy seeds
F. proliferatum (FS31)	83.33±11.78b	73.33±10.27b	0.34±0.02a	Rotted internally, discoloured
F. proliferatum (FS120)	61.66±10.27b	61.66±20.54b	0.32±0.00b	Rotted internally, discoloured
F. proliferatum (FS167)	53.33±4.71b	46.66±20.94c	0.30±0.00c	Rotted internally, discoloured
F. fujikuroi (FS79)	31.66±13.12c	61.66±10.27b	0.30±0.01c	Rotted externally, slightly discoloured
F. fujikuroi (FS101)	28.33±15.45c	60±8.16b	0.29±0.01c	Rotted externally, slightly discoloured
F. fujikuroi (FS123)	20±4.08cd	43.33±28.96c	0.35±0.02ab	Rotted externally, slightly discoloured
F. equiseti (FS4)	48.33±6.23c	16.66±6.23d	0.44±0.01a	Rotted externally, slightly discoloured
F. equiseti (FS65)	23.33±2.35c	18.33±4.7d	0.44±0.01a	Rotted externally, slightly discoloured
F. equiseti (FS170)	16.66±6.23cd	17.66±2.05d	0.34±0.15b	Rotted externally, slightly discoloured
F. acutatum (FS142)	100±0a	100±0a	0.30±0.03c	Rotted internally, fully discoloured
F. acutatum (FS151)	100±0a	100±0a	0.33±0.02b	Rotted internally, fully discoloured
F. acutatum (FS155)	100±0a	100±0a	0.32±0.02b	Rotted internally, fully discoloured
F. verticillioides (FS42)	100±0a	100±0a	0.32±0.01b	Rotted internally, fully discoloured
F. verticillioides (FS89)	96.66±4.71a	100±0a	0.29±0.01c	Rotted internally, fully discoloured
F. verticillioides (FS112)	100±0a	100±0a	0.36±0.00ab	Rotted internally, fully discoloured
F. incarnatum (FS2)	10±4.08e	31.66±13.12d	0.37±0.02ab	Rotted externally, slightly discoloured
F. incarnatum (FS24)	13.33±10.27e	17.66±5.24d	0.35±0.02ab	Rotted externally, slightly discoloured
F. incarnatum (FS130)	15±4.08e	26±5.35cd	0.38±0.00ab	Rotted externally, slightly discoloured
F. oxysporum (FS49)	58.33±11.78b	58.33±11.78c	0.31±0.01bc	Rotted internally, slightly discoloured
F. oxysporum (FS26)	66.66±11.78b	50±20.41c	0.33±0.02b	Rotted internally, slightly discoloured
F. oxysporum (FS132)	61.66±10.27b	33.33±11.78d	0.33±0.01b	Rotted internally, slightly discoloured
F. chlamydosporum (FS76)	31.66±13.12c	83.33±11.78b	0.37±0.02ab	Rotted internally, slightly discoloured
F. chlamydosporum (FS105)	33.33±10.27c	70±7.07b	0.37±0.00ab	Rotted internally, slightly discoloured
F. chlamydosporum (FS126)	36.66±8.49c	61.66±10.27b	0.33±0.02b	Rotted internally, slightly discoloured

Notes: The data mean average value from three independent replicates. Tukey's analysis was used to limit the significant difference at the level of P > 0.05. The different lowercase represents the significant differences of each parameter within the *Fusarium* species.

Table S8: Seed pathogenicity of *Colletotrichum* species isolated from intercropped soybean pods.

Isolates	PMC (%)	DSI (%)	Seed weight (g)	Seed symptoms
Control (CK)	0±0c	0±0e	0.36±0.03bc	Healthy seeds
C. fructicola (CS15)	100±0a	100±0a	0.35±0.0bc	Rotted internally, discoloured
C. fructicola (CS93)	83.33±6.23a	91.66±10.80a	0.33±0.02c	Rotted internally, discoloured
C. fructicola (CS169)	81.66±6.23a	85±11.78a	0.38±0.04b	Rotted internally, discoloured
C. truncatum (CS03)	98.33±2.35a	91.66±8.49a	0.45±0.01a	Rotted internally, discoloured
C. truncatum (CS61)	95±7.07a	83.33±4.71ab	0.43±0.02a	Rotted internally, discoloured
C. truncatum (CS153)	85±10.80a	83.33±11.78ab	0.45±0.05a	Rotted internally, discoloured

C. karstii (CS13)	51.66±6.23b	43.33±9.42c	0.42±0.04b	Rotted internally, discoloured
C. karstii (CS 96)	55±14.71b	51.66±6.23c	0.40±0.05b	Rotted internally, discoloured
C. karstii (CS 158)	60±12.24b	58.33±23.57c	0.34±0c	Rotted internally, discoloured
C. cliviicola (CS 22)	96.66±4.71a	70±7.07b	0.32±0c	Rotted internally, discoloured
C. cliviicola (CS95)	91.66±6.23a	61.66±12.47c	0.37±0.02bc	Rotted internally, discoloured
C. cliviicola (CS 164)	78.33±16.49a	45±14.71c	0.31±0.01c	Rotted internally, discoloured
C. plurivorum (CS06)	66.66±19.29b	38.33±10.27d	0.35±0.01bc	Rotted externally, slightly discoloured
C. plurivorum (CS172)	60±8.16b	35±10.80d	0.35±0bc	Rotted externally, slightly discoloured
C. plurivorum (CS180)	38.33±10.27b	33.33±11.78d	0.35±0.003bc	Rotted externally, slightly discoloured
C. boninense (CS94)	91.66±11.78a	75±12.24b	0.34±0.01bc	Rotted externally, slightly discoloured
C. boninense (CS118)	93.33±4.71a	81.66±13.12ab	0.31±0.03c	Rotted externally, slightly discoloured
C. boninense (CS166)	86.66±10.27a	66.66±11.78b	0.32±0c	Rotted externally, slightly discoloured

Notes: The data are the mean values from three independent replicates. Tukey's analysis was used to limit the significant difference at the level of $P > 0.05$. The different lowercase represents the significant differences of each parameter among *Colletotrichum* species.

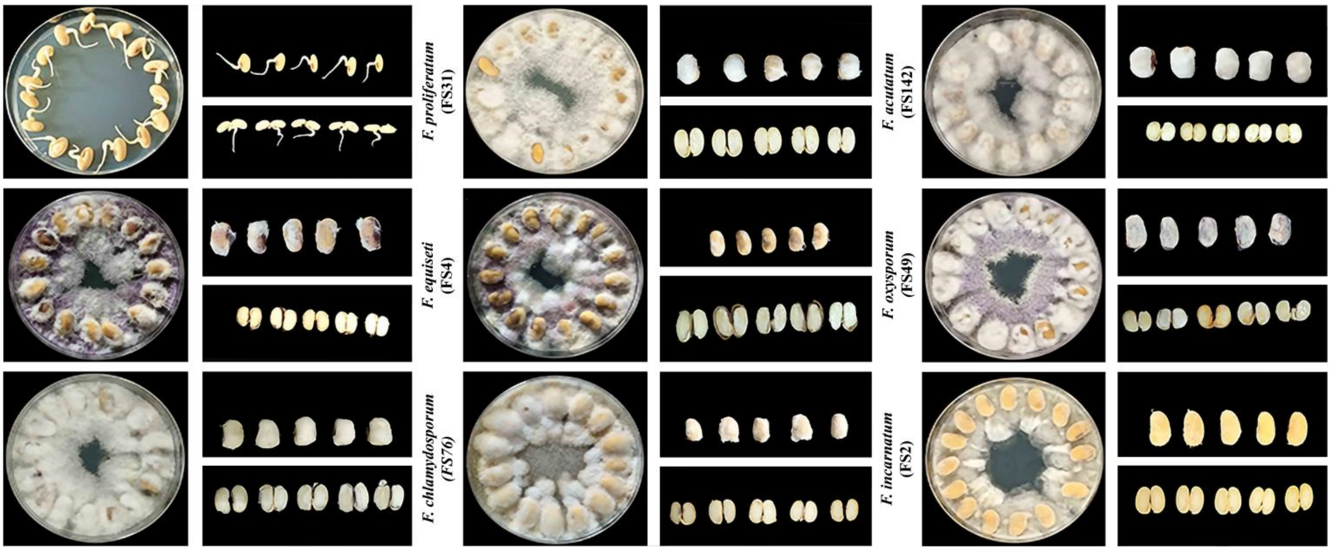

Figure S1: Seed pathogenicity of different *Fusarium* species isolated from intercropped soybean pods. Soybean seeds were inoculated with the representative *Fusarium* isolates through a seed soaking inoculation method at a final concentration of 1×10^5 spores per mL, and cultured on PDA medium. After 7 days of inoculation, disease symptoms were observed.

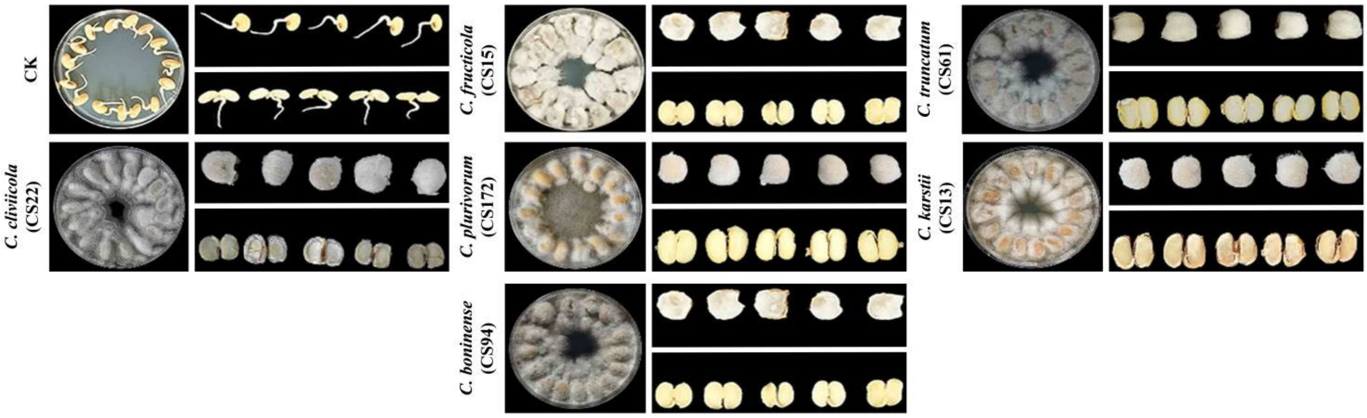

Figure S2: Pathogenic effects of different *Colletotrichum* species on soybean seeds. Soybean seeds were inoculated with the selected *Colletotrichum* isolates using a seed soaking method at a final concentration of 1 × 10^5 spores per mL placed and cultured on PDA medium. Disease symptoms were recorded after 7 days post-inoculation.

Disclaimer/Publisher's Note: The statements, opinions and data contained in all publications are solely those of the individual author(s) and contributor(s) and not of MDPI and/or the editor(s). MDPI and/or the editor(s) disclaim responsibility for any injury to people or property resulting from any ideas, methods, instructions or products referred to in the content.
